# A Delphi Survey on the Validity and Feasibility of a Healthcare-Associated Infection Surveillance System for Traditional Korean Medicine Hospitals in South Korea

**DOI:** 10.3390/healthcare13090991

**Published:** 2025-04-25

**Authors:** Sun Young Jeong, Ji Hye Park, Sung Eun Lee, Somi Shin, Kwan-Il Kim

**Affiliations:** 1 College of Nursing, Konyang University, Daejeon 35365, Republic of Korea; jsy7304@konyang.ac.kr (S.Y.J.); thal0624@naver.com (S.S.); 2 Department of Korean Medicine, College of Korean Medicine, Daejeon University, Daejeon 34520, Republic of Korea; jletter22@naver.com; 3 Infection Control Unit, Korea University Anam Hospital, Seoul 02841, Republic of Korea; covid19@kumc.or.kr; 4 Division of Allergy, Immune and Respiratory System, Department of Internal Medicine, College of Korean Medicine, Kyung Hee University Medical Center, Kyung Hee University, Seoul 02447, Republic of Korea

**Keywords:** Delphi method, healthcare-associated infections, infection control, surveillance, traditional medicine

## Abstract

**Background:** Current research on healthcare-associated infection (HAI) surveillance in traditional Korean medicine (TKM) institutions is limited. **Methods:** We utilized the Delphi method to evaluate the validity and feasibility of implementing an HAI surveillance system in TKM hospitals. This involved conducting a systematic literature review and focus group interviews with three infection control experts and five TKM doctors experienced in infection control within TKM hospitals. Based on these findings, we developed a Delphi questionnaire. The survey included a total of fifteen participants: ten TKM doctors and TKM-related policy researchers with infection control expertise, two infection control nurses, and three infectious disease doctors. **Results:** The survey results indicated strong consensus on the necessity of introducing an HAI surveillance system tailored to TKM hospitals, as well as their integration into the Korean National Healthcare-associated Infections Surveillance (KONIS) system. Since infectious diseases do not differentiate between acute care hospitals and TKM hospitals, it is reasonable for TKM hospitals to participate in infection surveillance systems. However, the feasibility of implementing HAI surveillance in TKM hospitals remains low due to a lack of awareness regarding infection surveillance, insufficient surveillance personnel, inadequate diagnostic and surveillance infrastructure, and limited policy support for infection control. Therefore, this study proposes a phased approach in which hand hygiene surveillance and safe injection practice monitoring, which received relatively higher consensus on feasibility, should be prioritized to establish the necessary surveillance infrastructure. Subsequently, a stepwise implementation of HAI surveillance can be introduced. **Conclusions:** Although TKM hospitals generally have a lower risk of HAIs compared to acute care facilities, they lack robust infection control systems and support. To address this gap, TKM hospitals should join the KONIS system. Appointing and training dedicated infection control personnel will enable their participation and enhance overall infection management.

## 1. Introduction

Infection control is a critical indicator for evaluating patient safety and quality of care. It is vital for protecting everyone within the hospital environment, including patients, healthcare workers, caregivers, and visitors [1]. Approximately 7–15% of patients in the acute phase of hospital care develop healthcare-associated infections (HAIs), leading to extended hospital stays, increased medical costs, and potential medical disputes [2]. For these reasons, managing HAIs is a crucial public health challenge worldwide [3], and the foundation of HAI management lies in establishing a national surveillance system to assess the current status of infections [4]. Such surveillance systems enable the establishment of key performance indicators and have been shown to be effective in reducing HAIs [5,6]. In South Korea, the Korean National Healthcare-associated Infections Surveillance (KONIS) system has been in operation since 2006. It initially focused on intensive care unit (ICU) infections but has gradually expanded to include surgical site infections, neonatal ICU infections, hand hygiene compliance, bloodstream infections, and long-term care hospital infection surveillance [7]. However, KONIS does not include traditional Korean medicine (TKM) hospitals, resulting in a lack of research on the infection control status in these institutions. TKM is a unique medical system in Korea, rooted in ancient Chinese medicine and incorporating treatments such as herbal medicine, acupuncture, moxibustion, cupping therapy, and Chuna manual therapy. TKM treatments are predominantly provided in outpatient settings rather than in hospitals, and TKM hospitals differ from acute care hospitals in that they rarely perform surgical or invasive procedures and have a limited use of antibiotics. Consequently, the risk of HAIs in TKM hospitals has generally been considered lower than that in acute care hospitals. However, since procedures such as acupuncture, cupping, and moxibustion involve direct stimulation of the skin, the possibility of infection cannot be completely ruled out.

Although cases of infection associated with TKM procedures are rare, such cases have been reported. For instance, the following cases of infection associated with TKM procedures have been reported: tuberculosis (TB) caused by unlicensed acupuncturists [8], methicillin-resistant Staphylococcus aureus infection after acupuncture treatment [9], 50 cases of acupuncture-related purulent skin infections [10], skin ulcers caused by non-tuberculous mycobacteria infection in South Korea and Canada [11], human immunodeficiency virus infection associated with acupuncture procedures in Thailand [12], and spondylitis [10]. Furthermore, a previous study has reported that 76% of TKM hospital workers have experienced stick injuries and 28.8% have had contact between mucosa and a patient’s blood or bodily fluids [13].

Even though the risk of HAIs in TKM hospitals is considered relatively low, certain procedures pose a higher risk of infection. Furthermore, given that patients are often transferred to TKM hospitals after receiving acute care treatment, infection control remains a crucial consideration. Moreover, as demonstrated during the coronavirus disease 2019 (COVID-19) pandemic, infectious diseases do not differentiate between hospital types. Therefore, integrating TKM hospitals into the national infection surveillance system is essential to ensure effective infection control.

Prior studies have investigated the knowledge and perceptions of infection control among TKM workers [14,15,16] and the state of infection control in TKM institutions [17]. However, research on surveillance of HAIs in TKM hospitals and the development of appropriate surveillance systems remains insufficient. In particular, no studies have assessed whether the KONIS system can be appropriately applied to TKM hospitals or if a separate surveillance system is required.

Therefore, this study aims to evaluate the validity and feasibility of introducing an HAI surveillance system in TKM hospitals using the Delphi method, which facilitates expert consensus. A panel of experts will be assembled to assess the necessity of infection surveillance in TKM hospitals, identify key surveillance targets, analyze barriers to implementation, and provide foundational data for establishing a surveillance system. Through this study, we aim to contribute fundamental data for future infection control policy development and improvements in infection surveillance systems.

Our specific objectives were as follows:

To analyze the validity of implementing an HAI surveillance system in TKM hospitals in South Korea.
To analyze the feasibility of introducing an HAI surveillance system into TKM hospitals in South Korea.

## 2. Materials and Methods

### 2.1. Study Design

The Delphi method is employed to study phenomena where data are sparse, obtaining objective results from the intuition of experts [18]. This systematic and structured technique uses a series of questionnaires to gather opinions and judgments from an expert panel. The responses are then aggregated to identify consensus opinions [19]. In our study, we applied the Delphi method to gather expert consensus on the validity and feasibility of introducing an HAI surveillance system for TKM hospitals.

### 2.2. Participants

Research indicates that a minimum of 10–15 experts is necessary to achieve significant results using the Delphi method [20]. To enhance the reliability and diversity of perspectives, we selected 18 experts with direct experience in HAI control and surveillance in TKM hospitals. The selection criteria were as follows: (1) TKM doctors or policy researchers with at least three years of experience in infection control within TKM hospitals, and (2) infectious disease specialists or infection control nurses with a minimum of five years of experience in hospital infection surveillance, particularly in the KONIS system. The Korean Medicine Hospitals’ Association (KOMHA) recommended 13 TKM experts, while the Korean Society for Healthcare-associated Infection Control (KOSHIC) recommended 5 infection control specialists. This ensured a balanced panel with expertise in both TKM-specific infection control and national-level infection surveillance systems.

### 2.3. Question Derivation

To develop preliminary questions assessing the validity and feasibility of introducing an infection surveillance system in TKM hospitals, we conducted a systematic literature review and focus group interviews (FGIs). We searched the PubMed (https://pubmed.ncbi.nlm.nih.gov/) (accessed on 21 August 2023), EMBASE (https://www.embase.com/) (accessed on 21 August 2023), Cochrane Library (https://www.cochranelibrary.com/) (accessed on 21 August 2023), and RISS (https://www.riss.kr/index.do) (accessed on 21 August 2023) databases. As search keywords corresponding to the central question, we included “TKM” and “TKM hospital” for the “participants” and “healthcare-related infection” and “healthcare-related infection surveillance system” for the “intervention”. As keywords for the search for “TKM” and “TKM hospital”, we selected the MeSH and Emtree terms of “Medicine, East Asian Traditional”, “Medicine, Korean Traditional”, “Acupuncture Therapy”, “Acupuncture”, “Moxibustion”, and “Cupping Therapy” as search terms. For “healthcare-related infection” we selected the MeSH and Emtree term “Cross Infection” as a search term, and for “healthcare-related infection surveillance system” we selected the MeSH and Emtree term “Safety Management” (Table 1).

The MeSH and Emtree terms corresponding to population (P) and intervention (I) were combined using “OR” to expand the research scope and include various relevant groups. Population (P) and intervention (I) were then combined using “AND” to comprehensively explore studies on HAIs that may occur in TKM hospitals and the surveillance systems for HAIs in these institutions.

The FGIs consisted of eight participants: five infection control managers or TKM doctors with at least three years of experience in infection control-related work or consulting at a TKM hospital, recommended by KOMHA, and three infection control doctors or nurses with at least five years of experience implementing the KONIS system. The FGIs explored various aspects of HAIs in TKM hospitals, such as the types, incidence, impact on patients and staff, and preventability. Discussions also addressed the necessity of TKM hospitals participating in the KONIS system and the types of infection surveillance required. Based on the findings of the FGIs, five major categories were identified: 1. HAIs in patients, 2. HAIs in staff, 3. process indicators for HAI surveillance, 4. outcome indicators for HAI surveillance, and 5. implementation of an infection surveillance system in TKM.

Based on the findings from the literature review and FGIs, we crafted a Delphi questionnaire focused on the validity and feasibility of implementing an HAI surveillance system in TKM hospitals. The survey consisted of 41 items evaluated on a 9-point scale and 2 open-ended questions. The 41 scaled items were designed to assess various aspects of HAIs and infection surveillance in TKM hospitals. Specifically, 21 items focused on the characteristics of potential HAIs, including 15 items evaluating the likelihood of occurrence, impact on patients, and preventability of infections such as skin and soft tissue infections (SSTs) following acupuncture, SSTs after bloodletting and wet cupping therapy, multidrug-resistant (MDR) bacterial infections, respiratory infections, and other skin infections. Additionally, six items assessed the likelihood, impact on staff, and preventability of occupational exposure to bloodborne infections and tuberculosis in TKM hospitals. The need and feasibility of implementing an infection surveillance system in TKM hospitals were examined through eight items evaluating the necessity and applicability of monitoring SSTIs, MDR bacterial infections, respiratory infections, and other skin infections. Four items assessed the necessity and feasibility of staff safety surveillance, focusing on occupational exposure to needlestick injuries and respiratory tuberculosis among TKM hospital staff. The survey also included six items examining the need and feasibility of process indicators in TKM hospitals, specifically monitoring hand hygiene, safe injection practices, and catheter-associated urinary tract infections. Lastly, two items evaluated the necessity of introducing an infection surveillance system in TKM hospitals. In addition to the scaled items, two open-ended questions explored barriers to TKM hospital participation in the national surveillance system and the requirements necessary for their integration. This survey structure was designed to systematically evaluate the validity and feasibility of implementing an infection surveillance system in TKM hospitals, reflecting expert consensus derived from the Delphi method.

### 2.4. Delphi Survey

The Delphi panel was formed by first requesting expert recommendations from the KOMHA and the KOSHIC, resulting in a list of 20 candidates. The purpose of the study was then explained via email, and a final panel of 18 experts was assembled based on their consent to participate.

We conducted the Delphi survey in two rounds, distributing questionnaires and collecting responses via email.

The first round took place from 15 December 2023, to 15 January 2024. The second round occurred from 7 February to 20 February 2024.

In the second round, to resolve discrepancies among panel members, participants were provided with their own responses from the first round alongside the panel-wide mean, median, content validity ratio (CVR), and qualitative responses. This process allowed participants to understand how their views compared with those of the entire panel and encouraged them to reassess or refine their stance on the validity and feasibility of introducing an HAI surveillance system in TKM hospitals, if necessary.

### 2.5. Data Analysis

We calculated the mean, median, and CVR for each survey response. The CVR was determined using the equation proposed by Lawshe [21]. The following equation was used:

$$\mathrm{CVR}=\frac{\mathrm{ne}-(\frac{N}{2})}{\frac{N}{2}}$$

where N is the number of responses, ne is the number of responses reporting agreement (corresponding to scores of 7, 8, or 9 on the 9-point Likert scale), and CVR defines a minimum value depending on the panel size. Responses with a CVR value above the minimum value were considered to show content validity. The panel size was 17 (first round) or 15 (second round) participants, and the minimum CVR was 0.49. Thus, responses with a CVR < 0.49 exhibit low content validity [21]. In this study, items were considered to have a high level of agreement if they met the following criteria: a mean score of 7 or higher, a median score of 7 or higher, and a CVR of 0.49 or above.

### 2.6. Ethics

The study protocol was reviewed and approved by the Institutional Review Board of Konyang University (KYU 2023-05-050-001). For the first Delphi survey, participants gave their consent via email, acknowledging that their participation was voluntary and that their anonymity would be maintained. They were informed that they could immediately request for their answers to be destroyed. The identities of the expert panel members were kept confidential throughout the data collection process, and questionnaires were distributed to each participant individually via email.

## 3. Results

### 3.1. Participant Characteristics

Of the 18 participants invited, 17 responded to the first round of the Delphi survey, achieving a response rate of 94.4%. From these, 15 participants continued to the second round, with a response rate of 88.2%. Among those who completed both rounds, the average age was 46.67 ± 8.58 years, and the male–female ratio was 46.7% male and 53.3% female. Regarding occupation, 46.7% were TKM doctors, 20.0% were researchers, another 20.0% were infectious disease doctors, and 13.3% were infection control nurses.

### 3.2. First Round

The first round surveyed 17 participants on their agreement regarding the validity and feasibility of introducing an HAI surveillance system for TKM hospitals (Table 2). Of the 41 responses, 15 had mean scores ≥ 7, 23 had median scores ≥ 7, and 10 had CVRs ≥ 0.49. Notably, responses to 10 questions (Questions 26, 30, 31, 34, 35, 36, 37, 38, 40, and 41) displayed a high level of agreement, with both mean and median scores ≥ 7 and CVRs ≥ 0.49.

#### 3.2.1. Characteristics of HAIs in TKM Hospitals

The responses concerning the likelihood, impact on patients or healthcare workers, and preventability of infections did not show a high level of agreement. These infections include skin or soft tissue infections following acupuncture treatments (such as general acupuncture, mini scalpel acupuncture, pharmaco-acupuncture, and needle-embedding therapy), skin or soft tissue infections after bloodletting and cupping therapy, multidrug-resistant (MDR) infections, respiratory infections, skin infections, and healthcare worker infections resulting from sharps injuries or exposure to tuberculosis in TKM hospitals. These responses generally had mean and median scores < 7 and CVR < 0.49. A summary of the Delphi panel’s qualitative responses indicated that infections related to acupuncture and cupping therapy are rare in TKM hospitals due to the widespread use of disposable needles and improved disinfection practices. However, MDR bacterial infections and respiratory infections were considered possible, though testing and isolation protocols were insufficient. While infectious skin diseases were reported to be uncommon, the risk of scabies transmission remains a concern, particularly among long-term inpatients. Additionally, needlestick injuries among staff were acknowledged as a potential risk, but the likelihood of subsequent infection was considered low. Although TB cases were infrequent, panelists noted that the screening and isolation measures for TB patients were inadequate. Overall, while the incidence of infections in TKM hospitals was perceived to be low, the panel emphasized the need for strengthened preventive measures and improvements in infection control systems.

#### 3.2.2. HAI Surveillance

Responses to Question 26 (“Respiratory infection surveillance is required for patient safety”) indicated a high level of agreement, with a mean score of 7.18, a median score of 7, and a CVR of 0.52. Participants emphasized the need for systems and support comparable to those in acute-care hospitals, including the implementation of diagnostic testing recommendations and inpatient wards during infectious disease outbreaks like COVID-19, as well as the need for quarantine procedure insurance. However, the responses to the question regarding the feasibility of surveillance for respiratory infections did not meet the criteria for agreement. Responses to questions on both the need for and feasibility of surveillance systems for infections met the criteria for agreement.

#### 3.2.3. Healthcare Worker Infection Surveillance

Responses to Question 30 (“Sharps injury surveillance is required for patient and worker safety”) demonstrated a high level of agreement, with a mean score of 7.35, a median score of 7, and a CVR of 0.53. Responses to Question 31 (“Sharps injury surveillance can be implemented”) also showed a high level of agreement (mean: 7.18; median: 7; CVR: 0.65). In response to Question 31, participants suggested that a sharps injury surveillance system could be integrated with other monitoring and feedback mechanisms, such as hand hygiene and injection procedures, and would be achievable with the availability of dedicated infection control staff.

#### 3.2.4. Infection Control Process Monitoring

Responses to Question 34 (“Hand hygiene surveillance is required for patient safety”) showed a mean score of 7.94, a median of 8, and a CVR of 0.88. Participants noted that hand hygiene is a highly cost-effective method of infection control. Responses to Question 35 (“Hand hygiene surveillance can be implemented”) also indicated a high level of agreement (mean: 7.59; median: 8; CVR: 0.65).

Responses to Question 36 (“Safe injection practice surveillance is required for patient safety”) revealed a mean score of 7.76, a median of 8, and a CVR of 0.88. Participants emphasized that process monitoring is crucial when administering medication at invasive sites. Responses to Question 37 (“Safe injection practice surveillance can be implemented”) showed a mean score of 7.41, a median of 7, and a CVR of 0.76. Here, participants recommended that a standardized surveillance system for injection practices be adapted and implemented to align with the unique characteristics of TKM hospitals.

Responses to Question 38 (“Catheter-associated urinary tract infection surveillance is required for patient safety”) had a mean score of 7.18, a median of 7, and a CVR of 0.53. Participants indicated that in TKM hospitals with inpatient wards, preventing urinary tract infections in patients with a Foley catheter is essential.

#### 3.2.5. Need to Introduce HAI Surveillance System

Responses to Question 40 (“There is a need to introduce an HAI surveillance system for TKM hospitals”; mean: 7.47; median: 8; CVR: 0.65) and Question 41 (“There is a need for TKM hospitals to participate in KONIS”; mean: 7; median: 7; CVR: 0.53) both indicated high levels of agreement. In response to Question 41, participants suggested that TKM hospitals should engage in infection surveillance systems because, as demonstrated by the recent COVID-19 outbreak, epidemics do not distinguish between acute-care and TKM hospitals. They emphasized the necessity for an understanding of KONIS system operations and the implementation of a dedicated infection surveillance system for TKM hospitals.

#### 3.2.6. Difficulties and Requirements for Participating in KONIS

In response to Question 42 (“Difficulties with participating in KONIS”), participants identified several obstacles to establishing a surveillance system: (1) a lack of infection control facilities, equipment, and personnel; (2) lack of infection surveillance personnel and support; (3) lack of HAI surveillance awareness—compared to acute-care hospitals, TKM hospitals conduct fewer invasive procedures and have lower infection control awareness; (4) regulatory constraints that impede participation in the KONIS system, with TKM doctors having limited authority on infection control; (5) the absence of infection surveillance criteria tailored to the specifics of TKM hospitals; (6) a deficiency in diagnosis and testing infrastructure; (7) a general lack of understanding among professionals specializing in HAI about the systems and procedures unique to TKM hospitals.

In response to Question 43 (“Requirements for participating in KONIS”), participants proposed the following needs: (1) funding for facilities, equipment, and personnel dedicated to infection control; (2) a surveillance system with operational support and improvements to infection control regulations and systems, including expanding the authority of TKM doctors to conduct infection tests and appointing them as infection control coordinators; (3) the development of a surveillance system that reflects the characteristics of TKM hospitals; (4) enhanced infection control awareness through education; (5) improvements to testing and diagnostic infrastructure.

### 3.3. Second Round

The second round of the Delphi survey was administered to 15 participants to assess the extent of agreement on the validity and feasibility of introducing an HAI surveillance system for TKM hospitals (Table 2). Responses to 13 out of 41 questions (round 1 results: 15) showed mean scores ≥ 7, and responses to 21 questions (round 1: 23) showed median scores ≥ 7. Responses to 15 questions (round 1: 10) showed CVRs higher than the criterion value of 0.49.

In the second round of the survey, responses to 13 questions (round 1 result: 10) had a high level of agreement, indicated by mean and median scores both ≥ 7 and CVRs ≥ 0.49. For responses to Question 26 (“surveillance of respiratory infections is required for patient safety”), which met all criteria (mean ≥ 7 points, median ≥ 7 points, CVR ≥ 0.49) in the first round, the mean level of agreement was lower in the second round (6.93) than the first round (7.18). On the other hand, compared to the first round, responses to an additional four questions met all the criteria in the second round (mean ≥ 7 points, median ≥ 7 points, CVR ≥ 0.49). The additional questions were Question 3 (“Skin and soft tissue infections after acupuncture treatment can be prevented”), Question 6 (“Skin and soft tissue infections after bloodletting therapy and cupping can be prevented”), Question 18 (“Sharps injuries can be prevented”), and Question 32 (“Respiratory tuberculosis exposure surveillance is required for patient and worker safety”).

Responses to Question 3 had a mean of 7.36, a median of 7, and a CVR of 0.6. Participants noted that while complete prevention of skin and soft tissue infections after acupuncture is challenging, proper sterilization before and after procedures, the use of disposable sterilized needles, gloves, and masks, and proper hand hygiene could significantly reduce infection rates. Therefore, monitoring adherence to these preventive measures during acupuncture is crucial.

Responses to Question 6 indicated a mean of 7.4, a median of 7, and a CVR of 0.87. Question 18 responses had a mean score of 7.2, a median of 7, and a CVR of 0.73. Participants suggested that investigating the types and causes of sharps injuries, as well as expanding efforts to include the prevention of infections caused by these injuries, could reduce the rate of sharps injuries.

Question 32 responses had a mean score of 7.0, a median of 7.0, and a CVR of 0.6. Participants emphasized that due to the potential presence of patients with tuberculosis in TKM hospitals, surveillance of tuberculosis exposure is crucial for ensuring the safety of patients and staff.

Through the two rounds of the Delphi survey, 13 items were identified as having a high level of agreement, meeting the criteria of a mean score of 7 or higher, a median score of 7 or higher, and a CVR of 0.49 or above. These included items assessing the preventability of SSTs after acupuncture, bloodletting, and cupping therapy (Items 3 and 6); the preventability of needlestick injuries among staff (Item 18); the necessity and feasibility of needlestick injury surveillance for staff (Items 30 and 31); the necessity of respiratory tuberculosis exposure surveillance for staff (Item 32); the necessity and feasibility of hand hygiene surveillance (Items 34 and 35); the necessity and feasibility of safe injection practice surveillance (Items 36 and 37); the necessity of catheter-associated urinary tract infection surveillance (Item 38); the necessity of implementing an infection surveillance system in TKM hospitals and integrating it into the national surveillance system (Items 40 and 41).

A narrative review of the Delphi items is presented in Supplementary Materials (Table S1).

## 4. Discussion

Based on the results of the systematic literature review and the FGIs, we formulated questions for a Delphi survey to assess the validity and feasibility of introducing an HAI surveillance system in TKM hospitals. From the literature and FGIs, we identified several potential HAIs in TKM settings, including: (1) skin or soft tissue infections following various types of acupuncture treatments (such as general, mini-scalpel, pharmaco-acupuncture, and needle-embedding therapies), (2) skin or soft tissue infections after bloodletting and cupping therapies, (3) MDR infections, (4) respiratory infections, (5) other skin infections, (6) healthcare worker infections due to sharps injuries, and (7) healthcare worker infections due to tuberculosis exposure.

For skin or soft tissue infections following acupuncture treatment, although the consensus on the likelihood and impact on patients did not meet all criteria, there was strong agreement on their preventability. Previous studies have highlighted that acupuncture procedures are a significant source of HAIs in TKM hospitals both globally and in South Korea. Reported complications from acupuncture include staphylococcal septicemia, laminar necrosis, streptococcal myositis [22], septicemia and compartment syndrome of the lower extremity [23], retroperitoneal abscess [24], and subcutaneous abscess caused by *S. aureus* [25]. Additionally, other HAIs associated with acupuncture in South Korea include infectious skin infections [26], chronic hepatitis [27], Mycobacterium fortuitum infections [28], spinal cord abscesses [29], cellulitis, and synovitis [30], as well as *M. abscessus* skin infections following needle-embedding therapy [31].

Participant feedback indicates that the risk of skin and soft tissue infections after acupuncture treatment has been low over the past five years due to rigorous disinfection practices and the use of single-use needles. However, the risk remains higher with more invasive procedures such as mini-scalpel acupuncture [32], pharmaco-acupuncture [33], and needle-embedding therapy [31]. Furthermore, the diagnostic criteria for identifying skin and soft tissue infections are not clearly defined, and infection rates are not routinely monitored, making accurate incidence estimates challenging. Therefore, further investigation into skin and soft tissue infections following these more invasive procedures is necessary.

For skin or soft tissue infections following cupping therapy, while the agreement on the likelihood and effects on patients did not meet all criteria, there was high consensus on their preventability. Previous studies have documented instances of herpes simplex virus infection [34] and blisters [35] resulting from cupping therapy. Although cupping can cause significant skin trauma, the risk of infection is minimal with proper disinfection practices and the use of single-use cups. Effective infection control measures, including consistent monitoring and feedback, can further reduce the infection rates in TKM hospitals by ensuring strict adherence to disinfection protocols and regulations.

Regarding MDR bacterial infections at TKM hospitals, there was low agreement on the likelihood, the effects on patients, and the preventability of such infections. While MDR bacterial infections can occur in patients transferred from acute-care hospitals to TKM hospitals for long-term care and rehabilitation, the risk is considered low because few patients at TKM hospitals are treated for infectious diseases and instances of antibiotic use and surgery are uncommon. However, the difficulty in performing diagnostic tests for MDR bacteria in TKM hospitals, combined with the absence of adequate response systems, means that the transmission of MDR bacteria could have severe consequences. Currently, TKM hospitals do not conduct diagnostic tests for MDR bacteria and the availability of isolation wards and equipment is limited, both of which pose challenges for infection prevention.

Regarding respiratory and skin infections in patients at TKM hospitals, there was also low agreement on the likelihood, effects on patients, and preventability. Although few patients are diagnosed with respiratory infections at admission, the risk of transmission increases without proper management. Given that elderly patients or those with underlying conditions are common in TKM hospitals, the effects of respiratory infections could be severe [36]. While infectious skin diseases are infrequent at TKM hospitals—typically requiring a dermatology consultation—early detection remains critical.

Regarding healthcare worker infections due to sharps injuries, the level of agreement on the likelihood and effects on workers was low, but agreement on preventability was high. Since bloodborne infectious diseases are rare in patients at TKM hospitals, the risk of contracting a disease or infection from a sharps injury is considered low. Nevertheless, implementing preventive measures and appropriate follow-up is crucial, as is ongoing education and management to ensure safety [14].

Regarding the necessity and feasibility of infection control process monitoring, there was high agreement on both the necessity and feasibility of hand hygiene and safe injection practices. Conversely, while there was high agreement on the necessity of monitoring for catheter-associated urinary tract infections, the feasibility of implementing such surveillance was considered low. Hand hygiene is crucial for reducing HAIs, and systematic monitoring of hand hygiene can help prevent these infections. Implementing hand hygiene monitoring in TKM hospitals is possible with proper education on hand hygiene surveillance systems and monitoring techniques. Although individual hospitals have endeavored to implement safe injection practices, the establishment of systematic monitoring could further help prevent HAIs. Designing and implementing a standardized injection practice surveillance system tailored for TKM hospitals is necessary. Urinary tract infection surveillance is crucial for patients in TKM hospitals who have catheters. However, implementation is challenging due to the limited authority of TKM doctors to prescribe diagnostic tests, a lack of testing systems, and a shortage of infection surveillance staff. The findings of this study indicate that infectious diseases do not distinguish between acute care hospitals and TKM hospitals, supporting the rationale for integrating TKM hospitals into infection surveillance systems. However, the feasibility of implementing infection surveillance in these hospitals was found to be low due to several challenges, including a lack of awareness regarding infection surveillance, insufficient surveillance personnel, inadequate diagnostic and surveillance infrastructure, and limited policy support for infection control. Given these constraints, a phased approach to infection surveillance implementation is recommended, starting with the monitoring of hand hygiene and safe injection practices, which received relatively high consensus on feasibility. Establishing a foundational surveillance infrastructure through these process indicators would enable the gradual expansion of infection surveillance in TKM hospitals. Additionally, to enhance the knowledge, awareness, and adherence to infection control practices among TKM hospital staff, the development and implementation of a structured training program tailored to the specific needs of TKM hospitals is proposed. Such initiatives would contribute to effectively strengthening the infection control capacity of healthcare professionals in TKM hospitals.

### Clinical Implications and Limitations

There was strong consensus on the need to introduce an HAI surveillance system in TKM hospitals in South Korea and the necessity for these hospitals to participate in KONIS, the ongoing national system, as evidenced in both rounds of the Delphi survey. Given that patients of TKM hospitals frequently use acute healthcare services, it is vital for TKM hospitals to be integrated into the national infection surveillance framework. A tailored infection surveillance system that reflects the specific practices and needs of TKM hospitals is essential, as is the establishment of dedicated infection control practitioners and departments to facilitate this integration. Inclusion of TKM hospitals could significantly enhance the effectiveness of national infection surveillance systems.

This study evaluated the feasibility and applicability of introducing an infection surveillance system in TKM hospitals through the insights of a 15-member Delphi panel, informed by a comprehensive literature review and feedback from eight expert focus group interviews. However, the generalizability of this study is limited due to challenges in including a diverse range of TKM hospitals across different regions and sizes, and varied experiences of TKM hospital professionals.

## 5. Conclusions

Patients and staff in TKM hospitals have a lower risk of HAIs compared to those in acute care facilities; however, infection control systems and institutional support remain significantly lacking. This study highlights the necessity of integrating TKM hospitals into the national infection surveillance system. However, establishing, operating, and evaluating an effective surveillance system requires proactive efforts from both TKM hospitals and public health authorities.

TKM hospitals must secure dedicated infection control personnel capable of conducting surveillance activities and ensure that they receive adequate support to effectively carry out infection prevention and control measures. Simultaneously, public health authorities should implement systematic strategies to facilitate infection surveillance and management in TKM hospitals. These efforts should include training programs for infection control personnel, development of a surveillance system tailored to TKM hospitals, and financial support for infection control activities.

Monitoring process indicators such as hand hygiene and safe injection practices is critical for patient safety and has been widely recognized as a feasible approach. Therefore, it is essential to establish practical surveillance standards and develop a structured surveillance system that reflects the unique characteristics of TKM hospitals.

Moving forward, progressive implementation of surveillance indicators for high-priority areas, informed by further research and development, will facilitate the effective integration of TKM hospitals into the national surveillance system. This stepwise approach will help ensure that infection surveillance in TKM hospitals is both feasible and sustainable, ultimately enhancing the overall infection control infrastructure.

## Figures and Tables

**Table 1:** Keywords and MeSH/Emtree terms for healthcare-associated infections and surveillance systems in traditional Korean hospitals.

PICO *	Keyword	MeSH and Emtrée Terms
P	TKM, TKM hospital	Medicine, East Asian Traditional, Korean medicine, Acupuncture Therapy, Acupuncture, Moxibustion, Pharmacopuncture, Cupping Therapy, Oriental Medicine
I	healthcare-related infection	Infections, Infection, Cross Infection
	healthcare-related infection surveillance system	Infection Control, Safety Management, Device Safety, Hygiene, Personal Protective Equipment, Protective Equipment

* P; participants, I; intervention, C; comparator, O; outcome.

**Table 2:** Results of a Delphi survey on the feasibility and applicability of introducing an infection surveillance system at traditional Korean medicine hospitals.

Category	No.	Question	Round 1 (*n* = 17)			Round 2 (*n* = 15)		
			Mean	Median	CVR	Mean	Median	CVR
Characteristics of HAI in TKM hospitals	1	At TKM hospitals, SSTs frequently occur after acupuncture treatment (e.g., general acupuncture, mini scalpel acupuncture, pharmaco-acupuncture, needle-embedding therapy).	4.00	3	−0.53	3.47	3	−0.87
	2	SSTs after acupuncture treatment have a serious effect on the patient.	4.18	3	−0.65	3.60	3	−0.73
	3 ^†^	SSTs after acupuncture treatment in TKM hospitals are preventable.	7.24	7	0.41	7.36	7	0.60
	4	In TKM hospitals, SSTs frequently occur after bloodletting and cupping therapy (wet cupping, etc.).	4.06	4	−0.76	3.80	4	−0.87
	5	SSTs after bloodletting and cupping therapy have a serious effect on the patient.	4.88	5	−0.65	4.73	5	−0.73
	6 ^†^	SSTs after bloodletting and cupping therapy in TKM hospitals are preventable.	7.06	7	0.29	7.40	7	0.87
	7	In TKM hospitals, MDR bacterial infections frequently occur (e.g., VRE; CRE).	3.88	4	−0.76	4	4	−0.73
	8	MDR bacterial infections have a serious effect on the patient.	6.35	7	0.18	6.80	7	0.20
	9	MDR bacterial infections in TKM hospitals are preventable.	4.76	5	−0.65	5.00	5	−0.73
	10	In TKM hospitals, respiratory infections (e.g., influenza, COVID-19) frequently occur.	6.06	6	−0.06	6.13	6	−0.33
	11	Respiratory infections have a serious effect on the patient.	5.88	6	−0.18	6.00	6	−0.47
	12	Respiratory infections in TKM hospitals are preventable.	5.94	7	0.06	6.2	6	−0.20
	13	In TKM hospitals, skin infections (scabies) frequently occur.	4.18	4	−0.53	4.13	4	−0.73
	14	Skin infections have a serious effect on the patient.	5.12	5	−0.53	4.87	5	−0.73
	15	Skin infections in TKM hospitals are preventable.	6.24	7	0.06	6.13	7	0.20
	16	TKM hospital healthcare workers frequently experience sharps injuries.	5.76	6	−0.06	5.93	6	−0.33
	17	Sharps injuries have a serious effect on the worker.	4.71	5	−0.65	4.53	5	−0.73
	18 ^†^	Sharps injuries in TKM hospitals are preventable.	7.18	7	0.41	7.20	7	0.73
	19	TKM hospital healthcare workers are frequently exposed to patients with tuberculosis.	4.29	5	−0.53	4.20	4	−0.87
	20	Worker exposure to tuberculosis patients has serious effects on workers and patients.	6.24	7	0.06	6.80	7	0.33
	21	Accidents involving *Mycobacterium tuberculosis* exposure in TKM hospitals are preventable.	5.47	5	−0.29	5.00	5	−0.60
HAI surveillance	22	SSTs surveillance ^‡^ after acupuncture treatment in TKM hospitals is required for patient safety.	6.35	7	0.06	6.73	7	0.07
	23	SSTs surveillance after acupuncture treatment in TKM hospitals can be implemented.	6.41	7	0.06	6.67	7	0.20
	24	MDR bacterial infection surveillance in TKM hospitals is required for patient safety.	6.59	6	−0.06	6.4	6	−0.20
	25	MDR bacterial infection surveillance in TKM hospitals can be implemented.	5.82	6	−0.41	5.6	6	−0.73
	26 *	Respiratory infection (e.g., influenza; COVID-19) surveillance in TKM hospitals is required for patient safety.	7.18	7	0.53	6.93	7	0.60
	27	Respiratory infection surveillance in TKM hospitals can be implemented.	6.12	6	−0.06	6	6.00	−0.47
	28	Skin infection (e.g., scabies) surveillance in TKM hospitals is required for patient safety.	7.06	7	0.41	6.87	7	0.20
	29	Skin infection surveillance in TKM hospitals can be implemented.	6.18	7	0.06	6.13	6	−0.20
Healthcare worker safety surveillance	30 *^,†^	Surveillance of sharps injuries at TKM hospitals is required for patient and worker safety.	7.35	7	0.53	7.27	7	0.87
	31 *^,†^	Sharps injury surveillance in TKM hospitals can be implemented.	7.18	7	0.65	7.20	7	0.87
	32 ^†^	Respiratory tuberculosis exposure surveillance in TKM hospitals is required for patient and worker safety.	7.00	7	0.29	7.00	7	0.60
	33	Respiratory tuberculosis exposure surveillance in TKM hospitals can be implemented.	5.76	5	−0.29	5.40	5	−0.60
Infection control process monitoring	34 *^,†^	Hand hygiene surveillance (monitoring and feedback) in TKM hospitals is required for patient safety.	7.94	8	0.88	8.27	8	1.00
	35 *^,†^	Hand hygiene surveillance in TKM hospitals can be implemented.	7.59	8	0.65	8.13	8	1.00
	36 *^,†^	Safe injection practice ^§^ surveillance (monitoring and feedback) in TKM hospitals is required for patient safety.	7.76	8	0.88	7.87	8	1.00
	37 *^,†^	Safe injection practice surveillance in TKM hospitals can be implemented.	7.41	7	0.76	7.47	7	1.00
	38 *^,†^	Catheter-associated UTI surveillance in TKM hospitals is required for patient safety.	7.18	7	0.53	7.2	7	0.87
	39	Catheter-associated UTI surveillance in TKM hospitals can be implemented.	6.94	7	0.41	6.93	7	0.60
HAI surveillance systems	40 *^,†^	There is a need to introduce an HAI surveillance system for TKM hospitals.	7.47	8	0.65	7.73	8	0.73
	41 *^,†^	There is a need for TKM hospitals to participate in KONIS.	7.00	7	0.53	7.27	7	0.73

* Responses with mean and median scores both ≥ 7 and CVR ≥ 0.49 in the first round of the Delphi survey. ^†^ Responses with mean and median scores both ≥ 7 and CVR ≥ 0.49 in the second round of the Delphi survey. ^‡^ investigating infection incidence and providing feedback. ^§^ e.g., skin disinfection, drug container surface disinfection, use of disposables, and separation of drug preparation and waste areas during pharmaco-acupuncture or other acupuncture procedures involving drug injections. COVID-19, coronavirus disease 2019; CRE, carbapenem-resistant *Enterobacteriaceae*; CVR, content validity ratio; HAI, healthcare-associated infection; KONIS, Korean National Healthcare-associated Infections Surveillance; SSTs, skin or soft tissue infections; TKM, traditional Korean medicine; UTI, urinary tract infection; VRE, vancomycin-resistant *Enterococcus.*

## Data Availability

No new data were created or analyzed in this study.

## Supplementary Materials

The following supporting information can be downloaded at: https://www.mdpi.com/article/10.3390/healthcare13090991/s1, Table S1: Narrative comments from the results of the Delphi survey on the feasibility and applicability of introducing an infection surveillance system in traditional Korean medicine (TKM) hospitals.

## Abbreviations

The following abbreviations are used in this manuscript:

COVID-19	coronavirus disease 2019
CVR	content validity ratio
FGIs	focus group interviews
HAI	healthcare-associated infection
ICU	intensive care unit
KOMHA	Korean Medicine Hospitals’ Association
KONIS	Korean National Healthcare-associated Infections Surveillance
KOSHIC	Korean Society for Healthcare-associated Infection Control
MDR	multidrug-resistant
SSTs	skin or soft tissue infections
TKM	traditional Korean medicine
TB	tuberculosis

## References

[1] Jeong S.-Y., Oh H.-S., Chun H.-K. (2015). Analysis of the Status of Infection Controls After Application of the Healthcare Accreditation System. Korean J. Health Serv. Manag..

[2] World Health Organization (2011). Report on the Burden of Endemic Health Care-Associated Infection Worldwide.

[3] World Health Organization WHO Web Sites on Infection Prevention and Control. Health Care-Associated Infections. https://www.who.int/health-topics/infection-prevention-and-control.

[4] Pearson A. (2009). Historical and changing epidemiology of healthcare-associated infections. J. Hosp. Infect..

[5] Gastmeier P., Schwab F., Sohr D., Behnke M., Geffers C. (2009). Reproducibility of the surveillance effect to decrease nosocomial infection rates. Infect. Control. Hosp. Epidemiol..

[6] Choi J.Y., Kwak Y.G., Yoo H., Lee S.O., Kim H.B., Han S.H., Choi H.J., Kim Y.K., Kim S.R., Kim T.H. (2015). Korean Nosocomial Infections Surveillance System (KONIS). Trends in the incidence rate of device-associated infections in intensive care units after the establishment of the Korean Nosocomial Infections Surveillance System. J. Hosp. Infect..

[7] Lee M.S. (2021). Operation of the Nationwide Surveillance System for Healthcare-Associated Infection.

[8] Kim J.K., Kim T.Y., Kim D.H., Yoon M.S. (2010). Three Cases of Primary Inoculation Tuberculosis as a Result of Illegal Acupuncture. Ann. Dermatol..

[9] Woo P.C.Y., Lau S.K.P., Yuen K.-Y. (2009). First Report of Methicillin-Resistant Staphylococcus aureus Septic Arthritis Complicating Acupuncture: Simple Procedure Resulting in Most Devastating Outcome. Diagn. Microbiol. Infect. Dis..

[10] Woo P.C.Y., Lin A.W.C., Lau S.K.P., Yuen K.-Y. (2010). Acupuncture Transmitted Infections. BMJ.

[11] Xu S., Wang L., Cooper E., Zhang M., Manheimer E., Berman B., Shen X., Lao L. (2013). Adverse Events of Acupuncture: A Systematic Review of Case Reports. Evid. Based Complement. Alternat. Med..

[12] Panlilio A.L., Cardo D.M., Grohskopf L.A., Heneine W., Ross C.S. (2005). Updated U.S. Public Health Service Guidelines for the Management of Occupational Exposures to HIV and Recommendations for Postexposure Prophylax; US Centers for Disease Control and Prevention. https://www.cdc.gov/mmwr/preview/mmwrhtml/rr5409a1.htm.

[13] Callan A.K., Bauer J.M., Martus J.E. (2016). Deep Spine Infection After Acupuncture in the Setting of Spinal Instrumentation. Spine Deform..

[14] Shin H. (2019). Perception and Practice Level of Korean Medical Doctors on Infection Control and Prevention in Korean Medicine Facilities. J. Soc. Prev. Korean Med..

[15] Kim K.-M., Kim H.-J., Choi J.-S. (2012). Knowledge and Performance Level of Infection Control and Influencing Factors of Oriental Medical Doctors and Nurses in Korea. Korean J. Adult Nurs..

[16] Kim J., Sung S., Lee E. (2021). Perception and Comparison of Practice Level Depending on Infection Control Education by Korean Medicine Doctors. Korean J. Prev. Med..

[17] Kim K.M., Kim H.J. (2011). Nationwide Survey on the Current Status of Infection Control in Oriental Medical Hospitals. Korean J. Nosocom. Infect. Control..

[18] Hsu C.L., Sandford B.A. (2007). The Delphi technique: Making sense of consensus. Pract. Assess. Res. Eval..

[19] Boulkedid R., Abdoul H., Loustau M., Sibony O., Alberti C. (2011). Using and Reporting the Delphi Method for Selecting Healthcare Quality Indicators: A Systematic Review. PLoS ONE.

[20] Skulmoski G.J., Hartman F.T., Krahn J. (2007). The Delphi Method for Graduate Research. J. Inf. Technol. Educ. Res..

[21] Lawshe C.H. (1975). A Quantitative Approach to Content Validity. Pers. Psychol..

[22] MacPherson H., Lewith G.T. (2001). Reporting Adverse Events Following Acupuncture. Physiotherapy.

[23] Shah N., Hing C., Tucker K., Crawford R. (2002). Infected Compartment Syndrome After Acupuncture. Acupunct. Med..

[24] Cho Y.P., Jang H.J., Kim J.S., Kim Y.H., Han M.S., Lee S.G. (2003). Retroperitoneal Abscess Complicated by Acupuncture: Case Report. J. Korean Med. Sci..

[25] Woo P.C., Lau S.K.P., Wong S.S.Y., Yuen K.Y. (2003). Staphylococcus aureus Subcutaneous Abscess Complicating Acupuncture: Need for Implementation of Proper Infection Control Guidelines. New Microbiol..

[26] Song J.Y., Sohn J.W., Jeong H.W., Cheong H.J., Kim W.J., Kim M.J. (2006). An Outbreak of Post-acupuncture Cutaneous Infection Due to Mycobacterium abscessus. BMC Infect. Dis..

[27] Rim J.Y., Moon K.R. (2009). A Case of Chronic Hepatitis C Acquired Through Ear Piercing and Acupuncture. Korean J. Pediatr. Gastroenterol. Nutr..

[28] Choi Y.-J., Lee H.-J., Lee K.-Y., Ryu D.-J., Lee M.-G. (2009). A Case of Mycobacterium fortuitum Infection at the Site of Acupuncture. Korean J. Dermatol..

[29] Kim J., Kim S., Woo J.H. (2022). A Case of Deep Neck Infection and Spinal Cord Abscess Related with Acupuncture. Korean J. Otorhinolaryngol-Head Neck Surg..

[30] Do H.J., Lee E.J., Park G.H., Park Y.L., Seo J.C., Kim C.H., Yoon H.M. (2021). Cellulitis and Ankle Joint Synovitis After Acupuncture at BL60: A Case Report. Korean J. Acupunct..

[31] Hwangbo H., Moon S.-H., Jung S.-W., Lee S.-K. (2016). Mycobacterium abscessus Skin Infection Following the Embedding Therapy in an Oriental Clinic. Korean J. Dermatol..

[32] Cho K.H., Ki W., Yuk D.I., Sung I.S., Kim M.J., Hong K.E. (2013). Literature Study on the Infection Control of Dochim (刀鍼). Acupuncture.

[33] Lee M.-H., Son B.-W., Kim K.-M., Jeon S.-H., Kim Y.-K. (2018). A Study on Korean Traditional Medicine Side Effects Cases Described in Domestic Western Medical Journals in the Past 10 Years. J. Int. Korean Med..

[34] Jung Y.-J., Kim J.-H., Lee H.-J., Bak H., Hong S.P., Jeon S.Y., Ahn S.K. (2011). A Herpes Simplex Virus Infection Secondary to Acupuncture and Cupping. Ann. Dermatol..

[35] Na S.H., Seo Y., Shi H.J., Hwang I.S., Shin K.A., Son K.Y., Kim S.R., Shin M., Son H.J., Choi J.Y. (2025). A nationwide survey on infection prevention and control in acute care hospitals of Korea. J. Korean Med. Sci..

[36] Ministry of Health and Welfare. Results of the 2022 Korean Medicine Utilization Survey [Press Release]. 31 March 2023. https://www.mohw.go.kr/board.es?mid=a10503000000&bid=0027&tag=&act=view&list_no=375634&cg_code=.

