# Supplementary material for: A Delphi Survey on the Validity and Feasibility of a Healthcare-Associated Infection Surveillance System for Traditional Korean Medicine Hospitals in South Korea
Source: Healthcare (Basel). 2025 Apr 25;13(9):991. doi: 10.3390/healthcare13090991 (PMC12071445; doi:10.3390/healthcare13090991)
Supplement: Supplementary file 1 [file healthcare-13-00991-s001.zip › healthcare-3487192-supplementary.pdf]

Table S1. Narrative comments from the results of the Delphi survey on the feasibility and applicability of introducing an infection surveillance system in traditional Korean medicine (TKM) hospitals

Category	No.	Question	Narrative comments
Characteristics of HAI in TKM hospitals	1	At TKM hospitals, SSTs frequently occur after acupuncture treatment (e.g., general acupuncture, mini scalpel acupuncture, pharmaco-acupuncture, needle-embedding therapy).	There have been no documented cases of skin and soft tissue infections following acupuncture treatment in the past five years. The disinfection of treatment areas before and after acupuncture, along with the use of single-use needles, is a standard practice in oriental medicine clinics. Consequently, the probability of infection is relatively low. In general, acupuncture is less likely to cause infection in herbal hospitals that comply with infection control measures. However, invasive procedures such as acupuncture, medicinal acupuncture, and moxibustion should be handled with greater caution. Nevertheless, it is challenging to ascertain the actual frequency of skin and soft tissue infections due to the lack of clarity in the diagnostic criteria for skin infections and the absence of monitoring the infection rate.
	2	SSTs after acupuncture treatment have a serious effect on the patient.	Acupuncture can cause skin and soft tissue infections. These are usually not serious, but they can become more serious, such as osteomyelitis and muscle

		abscesses. Improvements in needles and disinfection have reduced the number of serious infections. There needs to be a way to assess how serious an infection is.
3#	SSTs after acupuncture treatment in TKM hospitals are preventable.	Acupuncture and moxibustion can cause skin inflammation, not infection. Infections from procedures can be prevented by following good infection control practices. In general, proper disinfection, using disposable needles, and wearing sterile gloves can minimize the occurrence of infections. Ensuring that your TKM hospital is implementing infection prevention activities and addressing any gaps can help reduce the incidence of infection.
4	In TKM hospitals, SSTs frequently occur after bloodletting and cupping therapy (wet cupping, etc.).	Due to the nature of the procedure, skin damage can be extensive, but with proper pre- and post-treatment disinfection and the use of disposable cups, infection is rare. Skin and soft tissue infections following wet cupping are rare, but may occur and should be monitored by the healthcare provider. Infection outbreaks are rarely reported but are considered likely and information sharing is needed to monitor and prevent infection.
5	SSTs after bloodletting and cupping therapy	The large area of invasion during wet cupping is likely to be more symptomatic

	have a serious effect on the patient.	if infection occurs, but few infections have been reported. The incidence of skin and soft tissue infections is decreasing due to pre- and post-treatment disinfection, and the criteria for severe effects are not clear and need to be evaluated. Infections following cupping may have more severe effects than acupuncture, but this is difficult to assess as they are rarely reported.
6#	SSTs after bloodletting and cupping therapy at TKM hospitals are preventable.	Despite infection control, soft tissue inflammation can occur. Prevention is possible with current disinfection, use of disposable cups and sterile gloves. Infection is preventable with proper pretreatment and procedural protocols, and infection prevention measures should be thoroughly implemented to minimize soft tissue infections that occur after cupping. Evaluating whether infection prevention measures are sufficiently implemented in TKM hospitals and addressing gaps can reduce the incidence of infection.
7	In TKM hospitals, MDR bacterial infections frequently occur (e.g., VRE; CRE).	Although TKM hospitals rarely test for multidrug-resistant bacteria, it is estimated that there are many patients with multidrug-resistant bacteria due to the large number of patients transferred from acute hospitals for medical treatment and rehabilitation, but in reality few patients are treated for infectious

		diseases and few antibiotic treatments or surgeries, so the incidence of multidrug-resistant bacterial infections is expected to be low.
8	MDR bacterial infections have a serious effect on the patient.	Multidrug-resistant bacterial infections in TKM hospitals are generally infrequent, but when they do occur, they can have serious consequences due to the lack of a response system. Evaluation criteria based on patient characteristics in TKM hospitals are needed, and the exact frequency and severity of multidrug-resistant bacterial infections are unknown, making it difficult to assess.
9	MDR bacterial infections in TKM hospitals are preventable.	Prevention of multidrug-resistant bacterial infections may be difficult for the immunocompetent elderly, who are the main group of patients admitted to TKM hospitals, and the incidence may be lower in hospitals that focus on musculoskeletal diseases. Currently, the incidence of multidrug-resistant bacterial infections is low in TKM hospitals, but this may change in the future if the characteristics of inpatients change. For the surveillance and prevention of multidrug-resistant bacterial infections, medical doctors are authorized to perform tests, but TKM doctors are not. Currently, diagnostic tests for

		multidrug-resistant bacteria are not performed in TKM hospitals, and it is difficult to prevent infections due to the lack of isolation rooms and supplies.
10	In TKM hospitals, respiratory infections (e.g., influenza, COVID-19) frequently occur.	Respiratory infections occur at similar rates in TKM hospitals as in other medical institutions, regardless of the type of hospital. However, diagnosis by diagnostic tests is difficult. Most TKM hospitals operate multi-bed rooms, which facilitate the spread of respiratory infections, so it is necessary to exclude patients with respiratory symptoms from hospitalization.
11	Respiratory infections have a serious effect on the patient.	Respiratory infections, such as coronaviruses and influenza, can be transmitted between patients, hospital staff and patients, but are unlikely to have a serious impact with appropriate measures and treatment. However, in TKM hospitals, where many elderly patients with underlying medical conditions are admitted, respiratory infections can have a serious impact on patients and can spread easily if patient isolation and response systems are inadequate.
12	Respiratory infections in TKM hospitals are preventable.	Although it is possible to prevent respiratory infections in herbal hospitals through environmental management, mask use and hand hygiene, this is often difficult to achieve in practice because of the admission of elderly people and

		patients with underlying medical conditions. Prevention of infection is difficult in the current situation, and even when prevention guidelines are available in hospitals, they are often not fully implemented in practice, making transmission a problem.
13	In TKM hospitals, skin infections (scabies) frequently occur.	Infectious skin diseases were not common in TKM hospitals. The inpatient population of TKM hospitals needs to be reviewed. Scabies outbreaks and transmission can be a problem in TKM hospitals, especially if they have elderly patients staying for long periods in multi-bed rooms, as in long-term care hospitals.
14	Skin infections have a serious effect on the patient.	It is not common for scabies to make the patient worse, and there is treatment available to relieve the patient's symptoms, so it is unlikely to have a very serious impact. However, given the distress caused by symptoms such as itching and the difficulty of diagnosing and treating scabies quickly in an TKM hospitals, an outbreak of scabies can have a significant impact on the patient.
15	Skin infections in TKM hospitals are preventable.	Infectious skin diseases are rare in TKM hospitals, but if a patient is admitted with scabies, it can be treated and prevented with immediate measures.

		Prevention of infectious skin diseases relies on hygiene in patients' rooms, and dermatological care is needed to diagnose and treat scabies. Prevention of infection is difficult in the current situation, but prompt dermatological diagnosis, treatment and isolation are important and systems need to be put in place.
16	TKM hospital healthcare workers frequently experience sharps injuries.	Needlestick injuries are a common occurrence in TKM hospitals and can be caused by carelessness during acupuncture treatments or needling. Acupuncture needles are disposed of as disposable items in TKM clinics, which can lead to needlestick injuries, but the actual frequency of needlestick injuries is low and difficult to determine due to lack of monitoring.
17	Sharps injuries have a serious effect on the worker.	Even in the event of a needlestick injury, the likelihood of contracting a blood-borne disease is low due to the low prevalence of AIDS and other blood-borne infectious diseases among outpatients and hospitalized patients, and the likelihood of secondary infection after a needlestick injury is also unlikely due to the very small diameter of needles and syringes. In most cases, it is unlikely to have a serious impact on workers, but it is necessary to understand the actual

		situation regarding the frequency of infection following a needlestick injury, whether there are prevention activities and appropriate measures after an injury, and the frequency of infection following a needlestick injury.
18†	Sharps injuries in TKM hospitals are preventable.	Prevention of needlestick injuries can be achieved through ongoing training and management, and by taking precautions such as wearing gloves and ensuring proper disposal of disposable needles. Prevention of needlestick injuries is not only about preventing the needlestick injury itself, but also about preventing infectious diseases through appropriate pre- and post-incident measures, so "prevention of needlestick injuries" should be expanded to include "prevention of infectious diseases caused by needlestick injuries."
19	TKM hospital healthcare workers are frequently exposed to patients with tuberculosis.	Patients with latent TB are rarely seen in TKM hospitals and are unlikely to come into contact with TB patients with other illnesses. TB patients are difficult to detect in TKM hospitals because TB-specific tests are not widely used.
20	Worker exposure to tuberculosis patients has serious effects on workers and patients.	For outpatients, there is little impact from contact with TB patients, and for inpatients, the likelihood of exposure is very low because they can be screened through questionnaires and tests. Healthcare workers are screened for TB and

			wear masks to minimize the risk of exposure, so serious effects are unlikely. However, if there is actual exposure to a TB patient, the impact could be significant as workers could become ill with TB and there is potential for transmission.
	21	Accidents involving *Mycobacterium tuberculosis* exposure in TKM hospitals are preventable.	In TKM hospitals, inpatients can be screened for TB using questionnaires and basic tests, and workers can minimize the risk by wearing masks and undergoing regular TB screening. However, TKM hospitals generally do not perform chest X-rays or sputum tests on all inpatients or walk-in patients with respiratory symptoms, making detection difficult. TB symptoms are non-specific and difficult to diagnose, and it is difficult to determine whether a person has been exposed to M. tuberculosis, making it difficult to prevent exposure.
HAI surveillance	22	SSTs surveillance‡ after acupuncture treatment in TKM hospitals is required for patient safety.	Although the incidence of skin and soft tissue infections following acupuncture treatment in TKM clinics is relatively low, a surveillance system would be beneficial for patient and staff safety. It is necessary to establish diagnostic criteria and surveillance periods for skin and soft tissue infections in advance to

		monitor infection rates, which will enable effective infection prevention measures.
23	SSTs surveillance after acupuncture treatment in TKM hospitals can be implemented.	Surveillance of skin and soft tissue infections after acupuncture treatment in TKM hospitals is necessary, but is unlikely to be effective if applied generally. Surveillance targets, diagnostic criteria and duration of surveillance need to be discussed, and it is difficult to apply in outpatient-oriented Oriental medicine clinics, but it can be applied at hospital level, which requires an increase in infection surveillance staff. However, it is necessary to reassess the sustainability and cost-effectiveness of infection surveillance midway through the surveillance project.
24	MDR bacterial infection surveillance in TKM hospitals is required for patient safety.	The incidence of multidrug-resistant bacteria in TKM hospitals is considered extremely rare, so infection surveillance is not considered essential. In general, culture tests for multidrug-resistant bacteria are not performed in TKM hospitals. However, surveillance is necessary because patients are repeatedly admitted to and discharged from other hospitals. However, surveillance should

		not be limited to monitoring alone, but should be preceded by insurance coverage for isolation.
25	MDR bacterial infection surveillance in TKM hospitals can be implemented.	Limited diagnostic tests for monitoring multidrug-resistant bacteria in TCM hospitals make it difficult to identify infections. Requires coordination with medical departments. Difficult to implement in all TKM hospitals, limited to inpatients, and requires staff support to conduct surveillance. It also requires prescribing authority, interpretation of results, application of quarantine costs and special tests for infection surveillance. Considering these factors, infection surveillance will be difficult.
26*	Respiratory infection (e.g., influenza; COVID-19) surveillance in TKM hospitals is required for patient safety.	TKM hospitals need appropriate infection surveillance systems and support for the global epidemic of respiratory infections. TKM hospitals must have the mandate and authority for infection surveillance for respiratory infectious diseases. They must have the right to test for influenza and insurance coverage for isolation.
27	Respiratory infection surveillance in TKM hospitals can be implemented.	Although medical staff are available in TKM hospitals, it is difficult to monitor respiratory infectious diseases due to insufficient numbers and limited testing.

			There is a need for rapid and accurate tests for respiratory infectious diseases such as influenza, but tests cannot be prescribed by TKM doctors and there are restrictions on carrying out diagnostic tests.
	28	Skin infection (e.g., scabies) surveillance in TKM hospitals is required for patient safety.	As the incidence of skin infections in TKM hospitals is not very high and it is difficult to believe that it will have a serious impact on patients, it is difficult to believe that infection surveillance for skin infections is essential. However, if elderly patients are often admitted to multi-bed rooms for long-term care/rehabilitation, the outbreak of scabies may be a problem and infection surveillance may be necessary.
	29	Skin infection surveillance in TKM hospitals can be implemented.	Infection surveillance for scabies requires an understanding of the disease and prompt dermatological referral of suspected scabies patients. It is difficult to determine whether infection surveillance is feasible in situations where such a system is not available.
Healthcare worker safety surveillance	30*†	Surveillance of sharps injuries at TKM hospitals is required for patient and worker safety.	As acupuncture is one of the main treatments in TKM hospitals, monitoring for needle sticks is necessary for staff safety.

	31*†	Sharps injury surveillance in TKM hospitals can be implemented.	Surveillance activities for sharps injury are not difficult to carry out if there is a person in charge. However, for sharps injury, pre-accident prevention activities and post-accident measures are important and may be limited by the need for expert (medical) support.
	32†	Respiratory tuberculosis exposure surveillance in TKM hospitals is required for patient and worker safety.	Although exposure to active TB patients is unlikely in TKM hospitals, there is a risk and a surveillance system could be beneficial for patient and staff safety. However, health management and prevention of exposed staff requires measures such as reviewing chest X-ray results, which may be difficult to implement in TKM hospitals.
	33	Respiratory tuberculosis exposure surveillance in TKM hospitals can be implemented.	Exposure surveillance for respiratory TB is considered impractical because it requires specialized personnel and testing systems to diagnose patients with TB and to conduct post-exposure testing and monitoring of patients and staff exposed to TB.
Infection control process monitoring	34*†	Hand hygiene surveillance (monitoring and feedback) in TKM hospitals is required for patient safety.	Hand hygiene is essential to reducing healthcare associated infections in TKM hospitals and many infections can be prevented with systematic hand hygiene surveillance. Hand hygiene is one of the most important ways to reduce the risk

		of transmission and is a low-cost, effective infection control measure.
35*†	Hand hygiene surveillance in TKM hospitals can be implemented.	Hand hygiene monitoring can be easily implemented if the hand hygiene monitoring system and staff trained in hand hygiene monitoring are in place.
36*†	Safe injection practice§ surveillance (monitoring and feedback) in TKM hospitals is required for patient safety.	While many hospitals are already making efforts to ensure safe injection practices, more systematic surveillance, such as monitoring and feedback, would help to prevent healthcare-associated infections in hospitals. Given that injections are an invasive procedure involving the injection of drugs into an invasive site, we believe that monitoring for infections is most necessary.
37*†	Safe injection practice surveillance in TKM hospitals can be implemented.	The standardized system for monitoring injection practices needs to be adapted to the characteristics of TKM hospitals.
38*†	Catheter-associated UTI surveillance in TKM hospitals is required for patient safety.	In TKM hospitals, patients with urinary problems such as cancer surgery, severe gout, etc. will have indwelling catheters inserted or already have them in place, so it is necessary to monitor for indwelling catheter-related UTIs.
39	Catheter-associated UTI surveillance in TKM hospitals can be implemented.	It is considered that it is not easy to implement surveillance in practice because of the limited prescription of diagnostic tests by doctors of TKM and the need

			for specialists to identify indwelling catheter-associated urinary tract infections and a test system such as culture tests.
HAI surveillance systems	40*†	There is a need to introduce an HAI surveillance system for TKM hospitals.	I think TKM hospitals should have their own infection surveillance system. This would help patients who use other medical institutions like acute acre hospitals and long-term acre hospitals. To apply infection surveillance, insurance should cover screening, cultures, isolation, supplies, etc. and TKM doctors should be able to examine and prescribe.
	41*†	There is a need for TKM hospitals to participate in KONIS.	TKM hospitals should take part in the KONIS. They need an appropriate system that works for them. During the corona pandemic, we saw that infectious diseases affect everyone, including those who use TKM. TKM hospitals need to take part in the system. They should have a unique system that reflects how they treat patients. They also need an infection control officer and department.
	42	(Opinion) What are the challenges of participating in the Korean Healthcare - associated Infections Surveillance System	At present, the KDCA is conducting a study to determine the current status of infection control in medical institutions. As the government's will for infection control is strong, it is recommended that the infection control system in TKM

(KONIS)?	hospitals be included in the development plan for the promotion and development of TKM and receive support from the Ministry of Welfare for management items, incentives for excellent institutions, etc., and participate in KONIS to monitor and identify the current status.<br>The perception that the current system is working well without major problems, and uncertainty about the infrastructure, administrative costs, and appropriate compensation for the increased workload of infection surveillance that would result from the introduction of KONIS, are barriers to participation in KONIS.<br>Ensuring mandatory inspection rights and establishing a surveillance system that reflects the reality of TKM hospitals.<br>It is difficult to establish a practical surveillance system if there is no insurance coverage for isolation-related rooms, materials, diagnostic tests, etc.<br>It is necessary to support medical staff who can prescribe infection-control related prescriptions and evaluate test results in TKM hospitals and staff for infection-control activities.

43	(Opinion) What are the requirements for TKM hospitals to participate in the Korea Healthcare-sssociated Infections Surveillance System (KONIS)?	Establish a Patient and Staff Safety Management Committee. Established the "Infection Control Plan for TKM Facilities." Inclusion of TKM in the KDCA's comprehensive plan for the prevention and management of healthcare-associated secondary infections. Include infection control in medical institutions in the Fifth Five-Year Plan for the Development of TKM.<br>Establish a monitoring system (monitoring system taking into account the characteristics of TKM hospitals, compulsory training, etc.).<br>Establish a supervision system that reflects the reality of TKM hospitals<br>Establish a number that reflects the weak management status of TKM hospitals.<br>Provide support in advance for medical personnel who can prescribe infection control-related prescriptions and judge test results in TKM hospitals, as well as personnel for infection control activities, including insurance coverage for quarantine-related rooms, materials, diagnostic tests, and administrative support for participation in the surveillance system.

*Responses with mean and median scores both ≥7 and CVR ≥0.49 in the first round of the Delphi survey

†Responses with mean and median scores both ≥7 and CVR ≥0.49 in the second round of the Delphi survey

‡investigating infection incidence and providing feedback

§e.g., skin disinfection, drug container surface disinfection, use of disposables, and separation of drug preparation and waste areas during pharmaco-acupuncture or other acupuncture procedures involving drug injection

COVID-19, coronavirus disease 2019; CRE, carbapenem-resistant *Enterobacteriaceae*; CVR, content validity ratio; HAI, healthcare-associated infection; KDCA, Korea Disease Control and Prevention Agency; KONIS, Korean National Healthcare-associated Infections Surveillance; SSTs, skin or soft tissue infections; TKM, traditional Korean medicine; UTI, urinary tract infection; VRE, vancomycin-resistant *Enterococcus.*
